# Puerarin-Loaded Proniosomal Gel: Formulation, Characterization, *In Vitro* Antimelanoma Cytotoxic Potential, and *In Ovo* Irritation Assessment

**DOI:** 10.3390/gels12010072

**Published:** 2026-01-13

**Authors:** Sergio Liga, Andra Tămaș, Raluca Vodă, Gerlinde Rusu, Ioan Bîtcan, Vlad Socoliuc, Raluca Pop, Diana Haj Ali, Iasmina-Alexandra Predescu, Cristina Adriana Dehelean, Francisc Péter

**Affiliations:** 1 Faculty of Chemical Engineering, Biotechnologies and Environmental Protection, Politehnica University Timișoara, Vasile Pârvan No. 6, 300223 Timișoara, Romania; sergio.liga96@gmail.com (S.L.); raluca.voda@upt.ro (R.V.); gerlinde.rusu@upt.ro (G.R.); ioan.bitcan@upt.ro (I.B.); francisc.peter@upt.ro (F.P.); 2 Romanian Academy–Timişoara Branch, Center of Fundamental and Advanced Technical Research, Laboratory of Magnetic Fluids, 24 Mihai Viteazul Avenue, 300223 Timişoara, Romania; vsocoliuc@gmail.com; 3 Faculty of Pharmacy, “Victor Babeș” University of Medicine and Pharmacy Timișoara, Eftimie Murgu Square No. 2, 300041 Timișoara, Romania; pop.raluca@umft.ro; 4 University Clinic of Toxicology, Drug Industry, Management and Legislation, Faculty of Pharmacy, “Victor Babeș” University of Medicine and Pharmacy Timișoara, Eftimie Murgu Square No. 2, 300041 Timișoara, Romania; diana.haj-ali@umft.ro (D.H.A.); iasmina-alexandra.predescu@umft.ro (I.-A.P.); cadehelean@umft.ro (C.A.D.); 5 Research Center for Pharmaco-Toxicological Evaluation, “Victor Babeș” University of Medicine and Pharmacy Timișoara, Eftimie Murgu Square No. 2, 300041 Timișoara, Romania; 6 Renewable Energy Research Institute—ICER, Politehnica University Timișoara, Gavril Musicescu Street No. 138, 300501 Timișoara, Romania

**Keywords:** Puerarin, flavonoids, proniosomal gel, *in vitro* cytotoxicity, melanoma

## Abstract

Puerarin is a naturally occurring isoflavone with reported anticancer activity, yet its topical translation is constrained by limited stability and suboptimal dermal delivery. A Puerarin-loaded proniosomal gel was developed as a potential dermal delivery platform, and we performed an initial assessment of its antimelanoma activity and safety. The gel was produced by coacervation–phase separation using Span 60, Tween 80, phosphatidylcholine, and cholesterol. Physicochemical characterization included pH, entrapment efficiency, rheology, FTIR, DSC, and vesicle properties (DLS, PDI, ζ-potential). In silico geometry optimization and docking were carried out for melanoma-associated targets (MITF and DNMT3B). Biological effects were investigated *in vitro* on A375 melanoma cells using MTT, morphological analysis, and nuclear/mitochondrial staining, while irritation potential was evaluated *in ovo* by HET-CAM. The optimized formulation exhibited a skin-compatible pH and an entrapment efficiency of 62 ± 0.26%. DLS indicated a multimodal population, with a major number-weighted vesicle population in the 100–200 nm range, and a ζ-potential of −34.9 ± 0.14 mV. FTIR and DSC supported component incorporation without evidence of chemical incompatibility. The gel showed non-Newtonian, pseudoplastic, thixotropic flow, which is advantageous for topical use. Docking predicted meaningful affinities of Puerarin toward MITF and DNMT3B. The formulation reduced A375 viability in a dose-dependent manner (to 44.66% at 200 µg/mL) and, at higher concentrations, produced nuclear condensation and disruption of the mitochondrial network. HET-CAM classified the gel as non-irritant. The Puerarin-loaded proniosomal gel represents a promising topical platform with preliminary *in vitro* antimelanoma cytotoxic potential, warranting additional studies to validate skin delivery, efficacy, and safety.

## 1. Introduction

Skin diseases encompass a diverse spectrum of inflammatory, infectious, neoplastic, and wound-related conditions that collectively exert a substantial burden on global health [1,2]. Despite their heterogeneous nature, these disorders share a profound impact on patient quality of life, often requiring long-term management and posing challenges related to treatment safety and efficacy [3]. Beyond these dermatological diseases, cutaneous melanoma is one of the most aggressive forms of skin cancer, originating from melanocytes, and although it accounts for a small percentage of skin malignancies, it is responsible for most skin cancer-related deaths due to its high metastatic potential [4,5].

Traditional topical formulations (e.g., creams, ointments, lotions) often exhibit suboptimal drug delivery profiles, including: (i) poor penetration; (ii) rapid removal from the skin surface; and (iii) inconsistent therapeutic levels [6,7]. These limitations have directly stimulated interest in the development of advanced transdermal drug delivery systems (TDDS) capable of enhancing cutaneous penetration, prolonging residence time, and reducing systemic exposure [8,9]. Recent advances in nanotechnology and topical formulation science have yielded a new generation of delivery systems (e.g., liposomes, niosomes, proniosomes) reported to demonstrate dermal permeation and improved drug retention in the epidermis and dermis [10,11,12,13]. The emergence of novel gel-based delivery platforms (e.g., hydrogels, oleogels, nanogel systems) has introduced versatile and biocompatible formulations that can be engineered to enable controlled drug release and effective encapsulation of both hydrophilic and lipophilic compounds, thus improving stability, skin adhesion, and overall therapeutic performance in dermatological applications [14,15,16].

Recently, scientific research has focused on proniosomes, a new class of non-ionic surfactant-based vesicular systems that can be formulated as free-flowing powders or semisolid gels and, upon hydration, convert into niosomes (e.g., small unilamellar vesicles, 10÷100 nm; large unilamellar vesicles, 100÷3000 nm; multilamellar vesicles, ≥ 10 μm), capable of encapsulating both hydrophobic and hydrophilic bioactive compounds, thereby enhancing bioavailability and potentially improving therapeutic efficacy [12,17,18,19]. Structurally, proniosomes typically consist of a non-ionic surfactant (e.g., Span, Tween, Brij), cholesterol and phospholipids (e.g., phosphatidylcholine, lecithin), with vesicle formation, entrapment efficiency, and drug release kinetics being governed by factors such as HLB value, alkyl chain length and the surfactant/cholesterol ratio [19]. Especially for gel formulations intended for topical application, proniosomal systems fall into the class of transdermal nano-vesicular gels, which have been reported to improve skin permeation and provide a depot effect at the stratum corneum level, increasing the therapeutic efficacy of bioactive compounds while, at the same time, reducing systemic side effects [17,18,20]. In addition to formulation advances, melanoma management strategies are increasingly exploring safer, biologically targeted natural polyphenols. Flavonoids are of particular interest because they show multi-mechanistic anti-melanoma effects in experimental models, including inhibition of melanogenesis, suppression of melanoma cell proliferation and invasion, and promotion of apoptosis in experimental models [21,22]. Flavonoid-loaded nanocarriers have largely been focused on traditional vesicular systems (e.g., liposomes), but proniosomal gels are still scarcely reported, highlighting a significant gap in the current literature [23,24].

Within this expanding interest in flavonoids as multifunctional anti-melanoma agents, recent findings have also highlighted the therapeutic relevance of isoflavones. Puerarin (PUE), or 7-hydroxy-3-(4-hydroxyphenyl)-8-[(2S,3R,4R,5S,6R)-3,4,5-trihydroxy-6-(hydroxymethyl)oxan-2-yl]chromen-4-one, a naturally occurring isoflavone isolated from the dried roots of plants belonging to the *Pueraria* genus, is known to exert multiple biological activities including antioxidant, anti-inflammatory, cardioprotective, hepatoprotective, inhibitor of osteoclast activation and bone resorption, insulin resistance-attenuating, and neuroprotective effects [25,26]. Our recent study confirmed the potential anti-melanoma activity of PUE in A375 cells, while mitochondrial function remained within biocompatible ranges and LDH release was minimal across all concentrations tested, indicating a favorable cellular tolerance profile [27].

Therefore, this study aimed to formulate and physico-chemically characterize, for the first time, a PUE-loaded proniosomal gel. The obtained formulation was further evaluated for its cytotoxic potential *in vitro* on A375 human malignant melanoma cell line, as well as *in ovo* to identify its possible irritant potential for future topical applications.

## 2. Results and Discussion

The physicochemical properties and structural characteristics of the PUE-loaded proniosomal gel were systematically evaluated to confirm the successful formation of the vesicular system and to assess its potential as a topical delivery system. The optimized formulation was characterized through a comprehensive series of physicochemical analyses, including FTIR, DSC, DLS, ζ-potential and rheological evaluation, to investigate vesicle size distribution, surface charge, structural stability, and rheological behavior. In addition to these experimental assessments, computational studies involving geometry optimization and molecular docking were performed to evaluate the molecular conformation of PUE and its potential interactions with relevant melanoma biological targets. Furthermore, preliminary biological studies were accomplished, consisting of *in vitro* cytotoxicity and *in ovo* CAM assays, to explore the initial biological performance and safety profile of the selected formulation. The combined findings from these physicochemical, *in silico*, and biological investigations are presented and discussed in detail below.

### 2.1. Formulation of the Puerarin-Loaded Proniosomal Gel (PUE-ProN)

The formulation method applied in this study was designed based on previously reported proniosomal systems that demonstrated the effectiveness of nonionic surfactants in combination with cholesterol and phospholipids. Such studies have shown that proniosomal gels containing Span60, Tween80, cholesterol, and phospholipids can encapsulate poorly water-soluble compounds with high entrapment efficiency, forming stable vesicular systems upon hydration and potentially improving delivery performance [28,29,30].

In our study, the optimized proniosomal formulation was selected after screening multiple compositions and obtained by combining Span60, Tween80, phosphatidylcholine and cholesterol in proportions that ensured an appropriate balance between membrane rigidity and fluidity of the vesicle. The optimized composition of the PUE-loaded proniosomal formulation is presented in Table 1.

Span60 (Sorbitan monostearate, SP), a low-HLB nonionic surfactant, contributed to the formation of a rigid and cohesive bilayer structure [31,32]. Tween80 (Polyoxyethylene sorbitan monooleate, TW; high-HLB surfactant) was included to promote vesicle formation and reduce vesicle size [33,34], whereas cholesterol was used to improve bilayer packing and reduce membrane permeability, contributing to improved entrapment and physicochemical stability [35,36]. Phosphatidylcholine (PH) supported bilayer integrity and facilitated PUE incorporation within the membrane [37,38].

Together, these components acted synergistically to produce a stable proniosomal system with favorable physicochemical characteristics, yielding a PUE entrapment efficiency of 62 ± 0.26% (in the experimental conditions described in Section 4.2) and a physiologically acceptable pH of 7.1 ± 0.12.

### 2.2. Physicochemical Characterization of the Puerarin-Loaded Proniosomal Gel (PUE-ProN)

#### 2.2.1. Particle Size, Polydispersity Index and Zeta Potential Analysis

**Figure 1:**
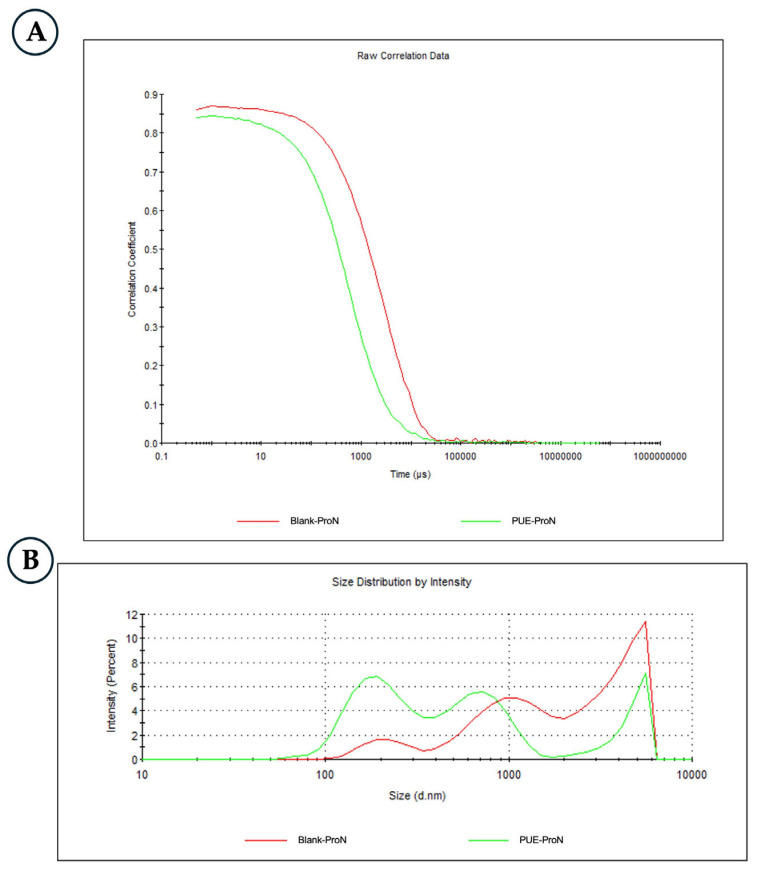
DLS analysis of Blank-ProN (red line) and PUE-ProN (green line): (**A**) correlogram data; (**B**) size distribution by intensity.

DLS analysis (Figure 1) performed on the vesicles obtained from the hydrated formulations showed that both the blank proniosomal gel (Blank-ProN, red) and the PUE-loaded gel (PUE-ProN, green) exhibited multimodal size distributions, together with high PDI values (0.705 ± 0.06 and 0.763 ± 0.17, respectively; *n* = 3), indicating pronounced polydispersity. After two weeks of storage, the short-term stability measurements for PUE-ProN indicated minimal change, with a PDI of 0.758 ± 0.04. The incorporation of PUE further increased the PDI, suggesting that the presence of isoflavone influences the structural organization of the vesicles during hydration and may promote the formation of additional aggregates under the tested preparation conditions [39,40]. The correlograms (Figure 1A) show Blank-ProN containing larger and more abundant PUE-ProN particles (vesicles and vesicle aggregates), while the distribution by intensity (Figure 1B) shows a predominant vesicle population in the 100–300 nm range together with a secondary contribution from vesicle aggregates (around 1000 nm). The number of aggregates is much smaller due to the quadratic dependence on particle volume of the scattered intensity. Also, Figure 1B shows that the Blank-ProN exhibits a higher degree of vesicle clustering than PUE-ProN.

Zeta potential (ζ-potential) values were −57.3 ± 0.11 mV for Blank-ProN and −34.9 ± 0.14 mV for PUE-ProN at neutral pH (*n* = 3). The observed ζ-potential trend can be attributed primarily to interfacial compositional changes driven by the Span/Tween ratio, which can modulate the electrokinetic slipping plane and ion adsorption at the surface, in concordance with other scientific reports [28,41].

#### 2.2.2. FTIR Analysis

**Figure 2:**
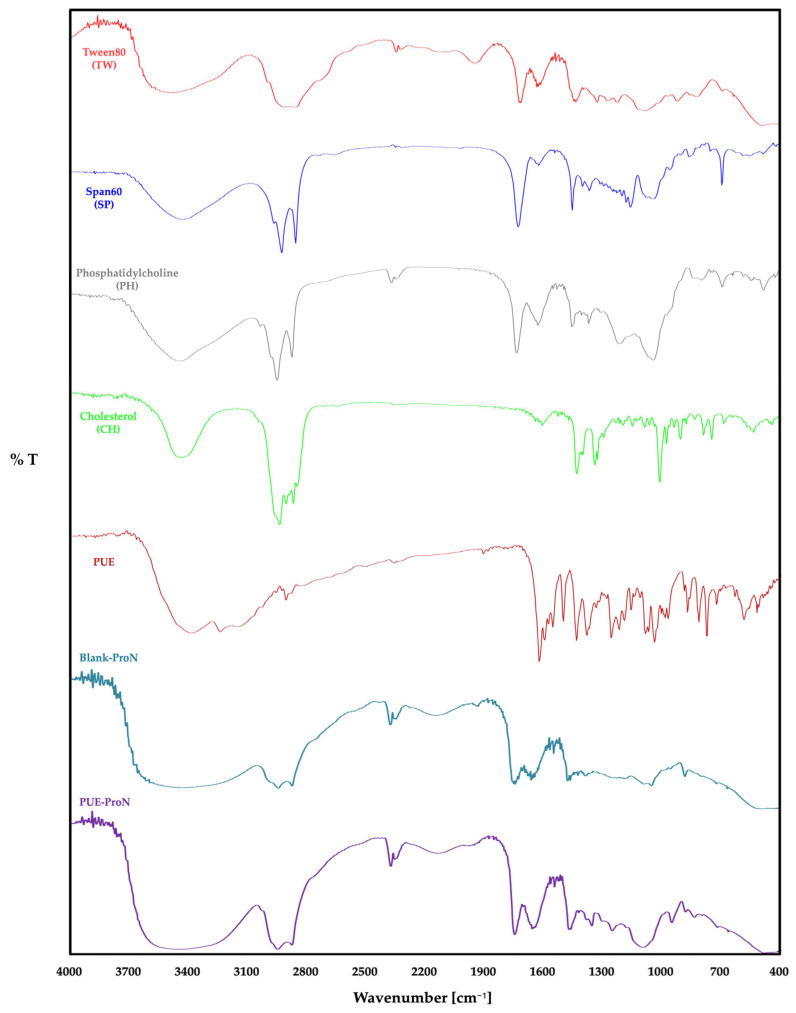
FTIR spectra of raw components (TW, SP, PH, CH), pure PUE, Blank-ProN and PUE-ProN.

Using FTIR spectroscopy analysis, potential chemical interactions between PUE and other components of the formulation were examined (Figure 2). The results of the FT-IR analysis of the PUE-ProN formulation, compared to the pure components PUE, SP, TW, PH, and CH, can be summarized as follows.

In the case of PUE, the characteristic absorption bands were observed at 3369 cm^−1^ and 3326 cm^−1^ corresponding to O-H stretching vibrations of phenolic groups, followed by a peak near 1633 cm^−1^ assigned to aromatic C=C stretching. Additional bands around 1550–1100 cm^−1^ represent -C-O vibrational stretching of the glycosidic linkage, while peaks near 1070–1030 cm^−1^ indicate C-O stretching of polyphenolic structures. SP exhibited characteristic peaks at 3427 cm^−1^ (O-H stretching), 2918 cm^−1^ and 2848 cm^−1^ (asymmetric and symmetric -CH_2_ stretching), and a sharp band near 1737 cm^−1^ corresponding to C=O ester stretching. TW showed prominent bands at 3516 cm^−1^ (O-H stretching), 2922 cm^−1^ and 2870 cm^−1^ (-CH_2_- asymmetric and symmetric stretching), and 1732 cm^−1^ representing C=O stretching of the ester group. CH exhibited characteristic peaks at 3431 cm^−1^ (O-H stretching), 2900–2850 cm^−1^ (C-H stretching of aliphatic CH_2_/CH_3_ groups), 1465 cm^−1^ (CH_2_ bending), and 1060–1030 cm^−1^ corresponding to C-O stretching vibrations. PH displayed typical phospholipid bands, including 2927 cm^−1^ and 2854 cm^−1^ (aliphatic -CH_2_ stretching), 1739 cm^−1^ (C=O ester stretching), and strong peaks near 1236 cm^−1^ and 1068 cm^−1^ attributed to PO_2_^−^ asymmetric and symmetric stretching vibrations.

The FTIR spectra of the Blank-ProN and PUE-ProN showed the characteristic broad O-H stretching band around 3200–3450 cm^−1^, indicating extensive hydrogen bonding within the surfactant-lipid matrix. The aliphatic -CH_2_/-CH_3_ stretching bands at 2918 cm^−1^ and 2852 cm^−1^, and the ester C=O band near 1737 cm^−1,^ confirmed the presence of Span 60, Tween 80, cholesterol, and phosphatidylcholine. For PUE-ProN, aromatic C=C and C-O bands between 1630 and 1500 cm^−1^ indicated the preservation of the PUE structure. The absence of new peaks or significant band deformation indicates that PUE was successfully encapsulated within the proniosomal matrix without undergoing chemical degradation or incompatibility with the components.

#### 2.2.3. DSC Thermal Analysis

**Figure 3:**
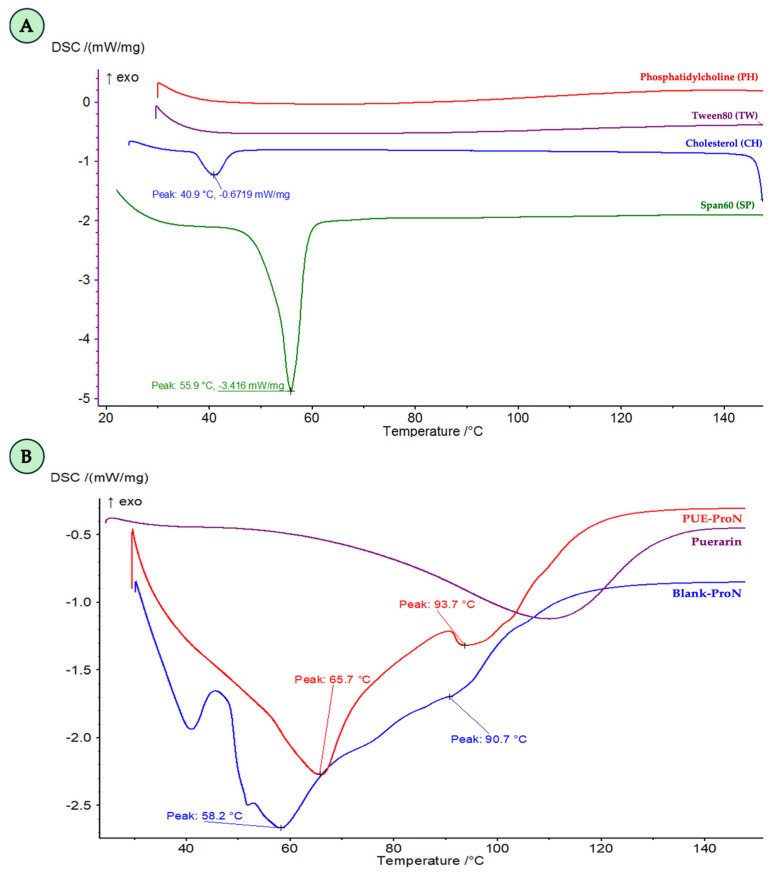
DSC thermograms of raw components (**A**) and PUE, Blank-ProN and PUE-ProN (**B**).

From the DSC curves, several endothermic transitions can be identified (Figure 3). The first major endothermic peak, appearing at approximately 60 °C, corresponds to the melting of the SP fraction (Figure 3A) and is clearly visible in both formulations. In addition to this main transition, a second endothermic event can be observed at higher temperatures (around 90–95 °C) in the gel matrices, suggesting further thermal reorganization of the surfactant-lipid assembly upon heating. Notably, the PUE-ProN trace displays a more pronounced baseline deviation, particularly after the primary melting transition. This thermal event may be associated with the loss of bound water or may reflect a solid-state polymorphic transition of the active ingredient PUE, as previously described in the literature [42].

#### 2.2.4. Rheological Characterization

**Figure 4:**
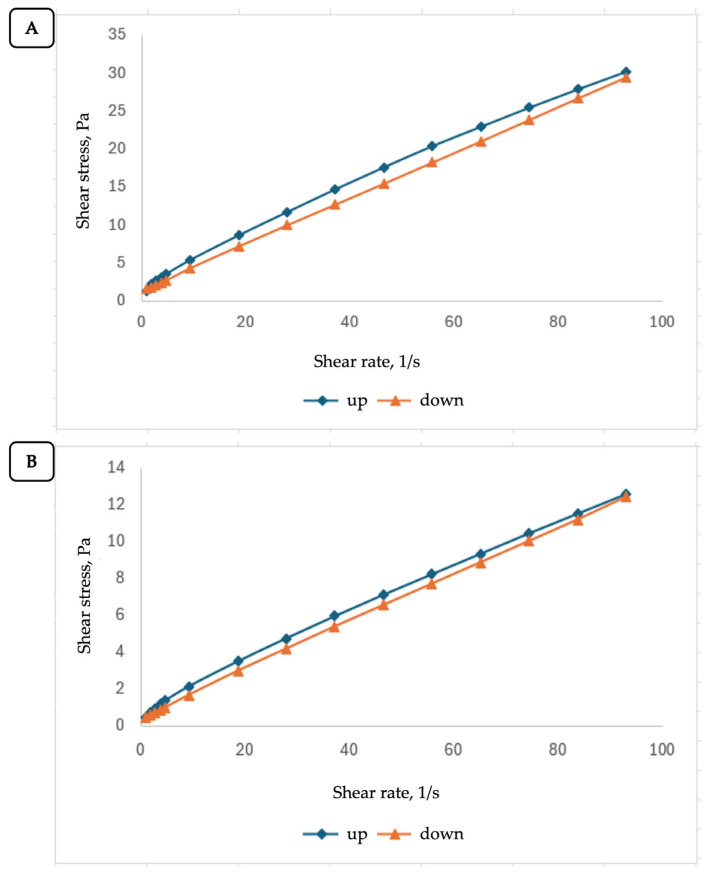
Flow profiles of the PUE-ProN sample at (**A**) 25 ± 0.5 °C and (**B**) 37 ± 0.5 °C.

For PUE-ProN, at 25 ± 0.5 °C and 37 ± 0.5 °C, the dependences between shear stress τ and shear rate $\dot{\gamma }$, are presented in Figure 4A,B, while the corresponding apparent viscosity and thixotropy values are summarized in Table 2.

The flow profiles at both temperatures indicated that our proniosomal gel behaves as a non-Newtonian, pseudoplastic (shear-thinning) fluid. The flow profiles may also show thixotropy behavior, which is associated with a hysteresis loop (the area between the shear-rate up and shear-rate down curves). The hysteresis loop shrinks with increasing temperature, approaching the shear stress values measured at the same shear rate.

For PUE-ProN, the Herschel–Bulkley parameters are presented in Table 3.

**Figure 5:**
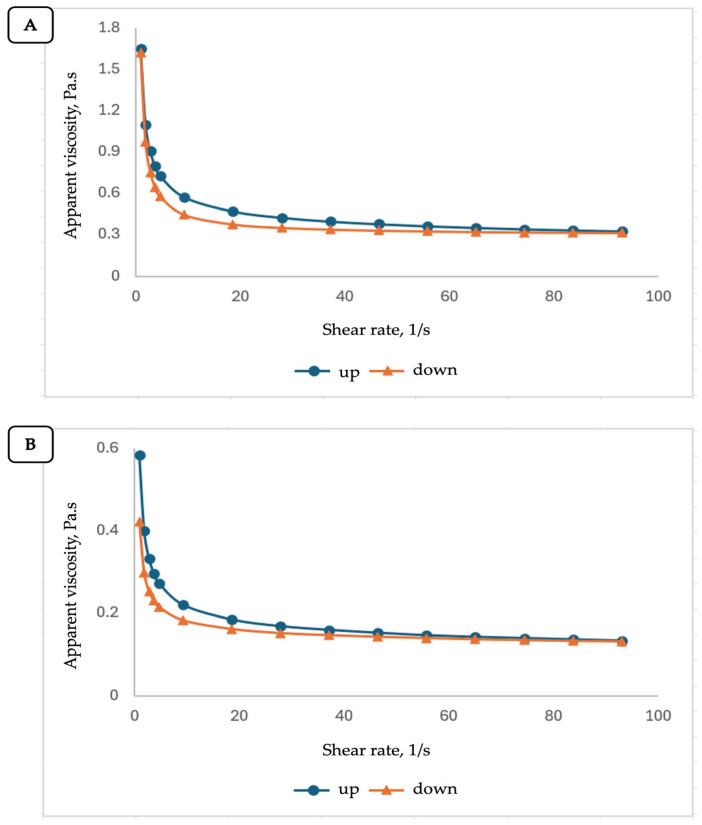
Viscosity profiles of the PUE-ProN sample at (**A**) 25 ± 0.5 °C and (**B**) 37 ± 0.5 °C.

Starting from Equation (2), the apparent viscosity values ($\eta_{a}$) were calculated by relating the shear stress to the shear rate. The dependences of apparent viscosity on shear rate are presented in Figure 5A,B and confirm the assumed thixotropic behavior of PUE-ProN, since at both temperatures the curve obtained at decreasing shear rate (down curve) is placed below the curve obtained at increasing shear rate (up curve). This suggests that the fluid becomes shear-thin with increasing shear rate and undergoes slower viscosity recovery as the shear rate decreases. Shear thinning is also confirmed, both from the dependence $\eta_{a}=f(\dot{\gamma })$ and from the subunit values of the flow index (*n*).

The results obtained for the PUE-ProN gel fit within the rheological behavior specific to these fluids. Thixotropic properties are particularly advantageous because they facilitate preparation, handling, and application to the skin, with the rapid formation of a stable and homogeneous film.

### 2.3. Computational Studies

To complement the experimental results and gain deeper insight into the molecular behavior of PUE, *in silico* computational modeling has been performed. These studies allow the evaluation of structural, electronic, and interaction-related properties that are not directly accessible experimentally but are essential to understand the reactivity and potential biological activity of PUE.

Geometric optimization has been performed both in the gas phase and the aqueous phase. The optimization in the presence of the solvent leads to structures that are more physically accurate and may lead to differences regarding the orientation of the hydroxyl groups capable of hydrogen-bonding.

**Figure 6:**
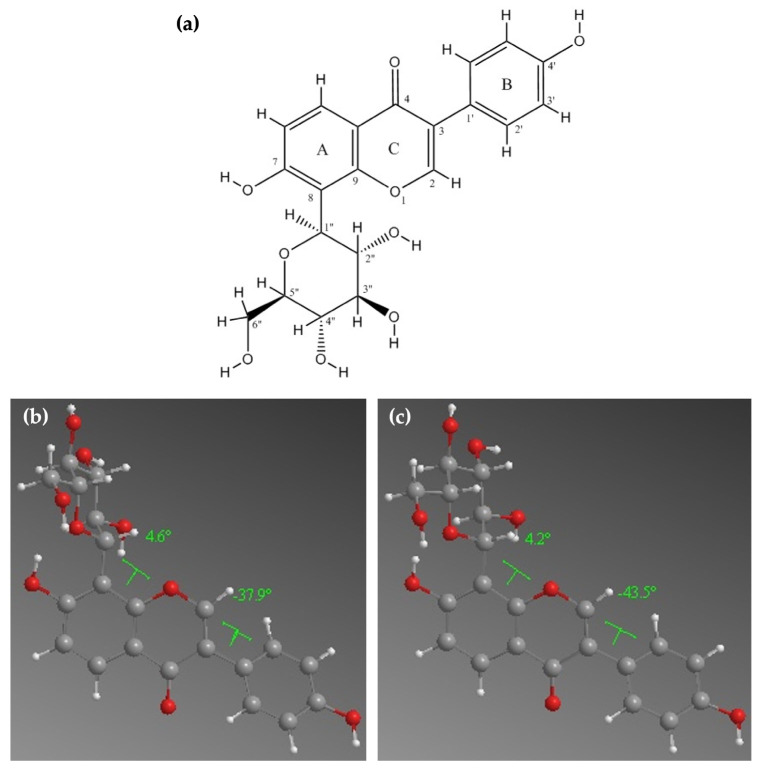
The atom numbering (**a**), gas-phase (**b**) and aqueous (**c**) optimized structures of PUE.

The optimized gas- and aqueous phase structures of PUE, along with the calculated dihedral angles between the chromen-4-one and phenolic (AC-B), respectively, chromen-4-one and glucose (AC-glucose) rings, are presented in Figure 6.

According to the geometries presented in Figure 6b,c, the main difference between the PUE (gas) and PUE (aq.) consists in the values of the dihedral angle formed by the chromene and phenyl rings. Closer values for the dihedral angle have been obtained for the chromene and sugar moiety, respectively.

Based on the determined geometries, the calculated steric parameters Connolly Accessible Area (CAA), Connolly Solvent-Excluded Volume (CSEV), and ovality have led to almost equal values for ovality and CSEV. The slightly different arrangement of the benzopyran and phenyl cycles of PUE (aq.) has led to a small increase in the CAA parameter (Table 4).

The systemic antitumor potential of PUE was evaluated through molecular interaction studies involving two relevant melanoma-associated targets (e.g., microphthalmia-associated transcription factor—MITF, 4atk.pdb; DNA methyltransferase 3B—DNMT3B, 5ciu.pdb). The structure of the receptor has been downloaded from RCSB Protein Data Bank [43]. Two key molecules were chosen as reference: ML329 as MITF inhibitor and 5-azacytidine as DNMT3B inhibitor.

#### 2.3.1. Docking-Based Prediction of PUE as a Potential MITF Inhibitor

**Figure 7:**
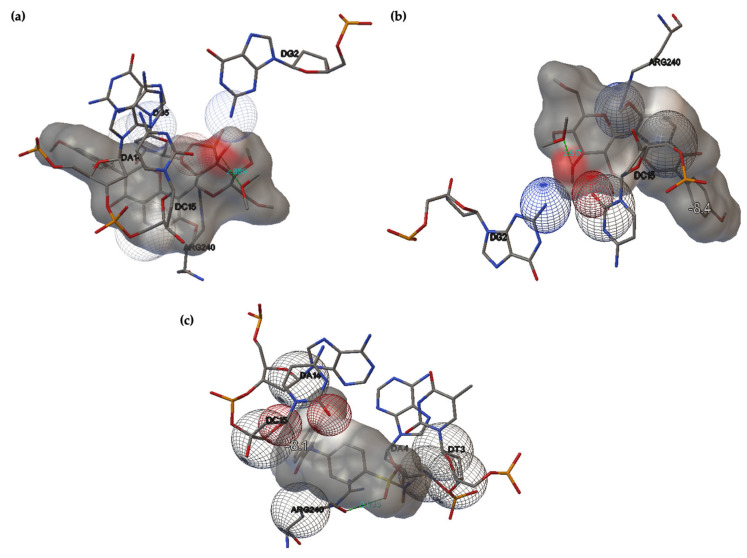
Interactions between PUE (gas-phase)-MITF (**a**), PUE (aq.)-MITF (**b**), and reference ML329-MITF (**c**).

For the specific case of melanoma, MITF acts as an oncogene, promoting the survival and proliferation of cancer cells. In addition, MITF also regulates melanocyte differentiation, which can promote malignant behavior. This dual role, in particular its involvement in the progression of melanoma and its link to therapeutic resistance, makes MITF a complex and critical factor in melanoma biology [44]. The binding affinities of PUE-MITF are presented in Table 5, while their interactions are presented in Figure 7 and summarized in Table 6.

The only amino acid residue with which PUE interacts is Arg240, a key amino acid from the basic region of MITF [45,46]. For both the gas-phase and aqueous optimized PUE, a close-contact interaction between the chromene skeleton and Arg240 is obtained. A larger number of interactions are established with the DNA nucleosides: deoxycitosine DC15, deoxythymidine DT16, deoxyguanosine DG2 and DG5, deoxyadenosine DA14. For both PUE (gas) and PUE (aq.), hydrogen bonds are obtained through the interaction of sugar hydroxyl groups 3″-OH and 4″-OH and DT16, respectively. The higher number of interactions with nucleobases may suggest that the ligand PUE is selective for specific DNA regions [47]. Comparison with ML329, a known inhibitor of MITF, show similar behavior to PUE: the same residue DT16 involved in the formation of the hydrogen bond, and hydrophobic interactions with the residue Arg240 and the DNA nucleosides DC15 and DA14. The lower values of the binding affinities calculated for ML329 can be attributed to its smaller calculated values of the steric parameters CAA, CSEV and ovality (532.70 Å2, 237.101 Å3, and 1.480, respectively).

#### 2.3.2. Docking-Based Prediction of PUE as a Potential DNMT3B Inhibitor

**Figure 8:**
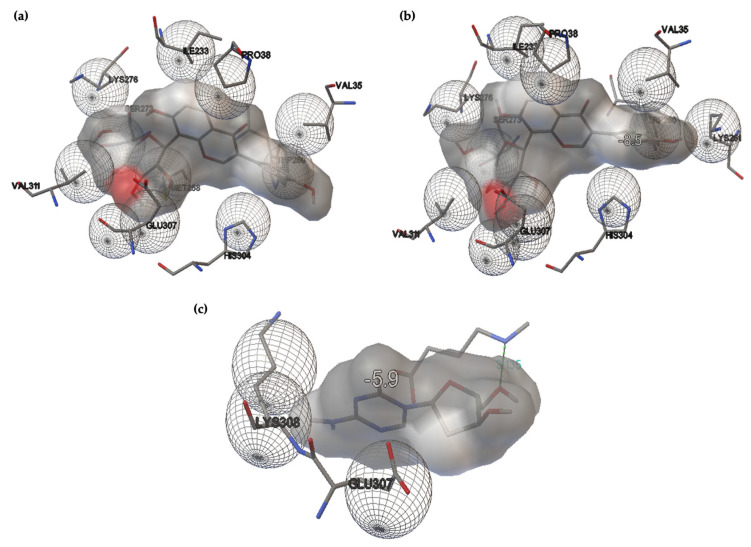
Interactions between PUE (gas-phase)–DNMT3B (**a**), PUE (aq.)-DNMT3B (**b**) and reference 5-azacytidine-DNMT3B (**c**).

Mutations in the DNMT3B gene are associated with immunodeficiency and centromere instability, while its abnormal activity contributes to cancers by altering the methylation of tumor suppressors and other key genes [48]. In cancer, aberrant DNMT3B activity can lead to the hypermethylation (silencing) of tumor suppressor genes, contributing to tumor development [49]. DNMT3B is frequently overexpressed in melanoma and functionally contributes to tumor initiation and progression, primarily through its role in the deregulation of DNA methylation and the repression of key tumor-suppressive pathways, as highlighted in recent research on melanoma [50]. Table 7 shows the calculated binding affinities of PUE towards DNMT3b; their interactions are presented in Figure 8 and listed in Table 8.

The interactions with the DNA methyl transferase 3B (DNMT3B) show that PUE binds to three of the receptor key amino acids Ile233, Pro38 and Val35 [50]. In addition, PUE shows an interaction with the Lys276 residue, which corresponds to the positively charged surface of the DNMT3B PWWP domain [51]. It can be observed that all three cycles that make up the PUE structure are involved in interactions with some of the key residues of the DNMT3B receptor. Although hydrogen bonds have not been formed, the large number of hydrophobic interactions leads to the good binding affinities that have been calculated for both PUE-optimized structures. Comparison to the hypomethylating agent 5-azacytidine shows a difference in the behavior of the compounds: the reference azacitidine establishes one hydrogen bond with the residue Glu271 and two hydrophobic interactions with Glu307 (which also interacts with PUE) and Lys308. It may be observed that PUE interacts with a larger number of key amino acids of the receptor, which is outlined by the larger values of its binding affinities. Also, it must be stated that significant differences appear in the geometry of the compounds; the steric parameters calculated for azacitidine are 408.205 Å2, 173.278 Å3, and 1.322 (CAA, CSEV and ovality, respectively). The smallest number of interactions between azacitidine and the considered receptor may be due to the above-mentioned structural differences.

### 2.4. In Vitro Cytotoxic Evaluation of PUE-ProN

#### 2.4.1. Cell Viability Assessment Using the MTT Method

The *in vitro* study started with the evaluation of PUE, Blank-ProN, and PUE-ProN on A375 cells in terms of cell viability after the application of the treatment regimen for 24 h. Cytotoxicity was determined using the MTT (3-[4,5-dimethylthiazol-2-yl]-2,5-diphenyltetrazolium bromide) assay, a colorimetric method that measures cell viability by assessing metabolic activity. The principle of this technique is based on the ability of living cells to enzymatically reduce the tetrazolium salt MTT into insoluble formazan. The quantity of formazan formed is directly correlated with the number of metabolically active cells, providing an indirect indication of cell viability [52].

**Figure 9:**
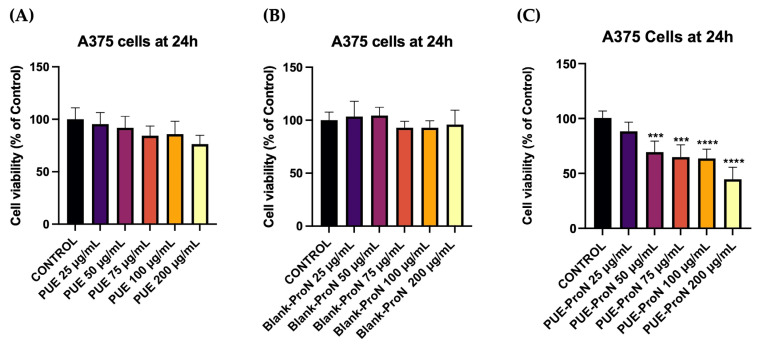
The evaluation of the *in vitro* cell viability after 24 h of treatment with PUE (**A**), Blank-ProN (**B**) and PUE-ProN (**C**) at 25, 50, 75, 100, and 200 µg/mL in the A375 cell line. Results are expressed as viability percentages (%) normalized to control (untreated cells). Presented data are expressed as mean values ± SD of three independent experiments performed in triplicate. A one-way ANOVA test was performed to observe the statistical differences between the control and the treated group, and then Dunnett’s multiple comparisons post hoc test was performed. “*” indicates statistical significance (*** *p* < 0.001, **** *p* < 0.0001).

To evaluate the cytotoxic potential of the proniosomal gel, the active ingredient PUE was tested at concentrations of 25, 50, 75, 100, and 200 µg/mL. It exhibited a moderate, concentration-dependent reduction in cell viability (Figure 9A). At lower doses (25–50 µg/mL), the viability remained close to the control values (95.38% and 91.9%), indicating minimal cytotoxic impact. A more noticeable reduction was observed at intermediate concentrations, with viability decreasing to 84.28% and 85.74% at 75 and 100 µg/mL, respectively. At the highest concentration tested (200 µg/mL), PUE reduced more substantially the cell viability, reaching 76.36% and suggesting a moderate cytotoxic effect at highest doses. Overall, these results indicate that PUE induces a mild-to-moderate, dose-dependent cytotoxic response.

Given that the formulation vehicle itself can influence cellular metabolic activity, the current study first evaluated the effect of the Blank-ProN. The results showed that Blank-ProN did not exert cytotoxic effects on cells at any of the concentrations tested (Figure 9B). A slight stimulatory effect was observed at lower concentrations, with cell viabilities of 103.42% and 104.47% at 25 and 50 µg/mL, respectively. At higher concentrations (75–200 µg/mL), the viability remained close to that of the control, with values of 93%, 92.28%, and 95.82%. Overall, these results confirm that the proniosomal vehicle is biologically safe and does not interfere with cell metabolic activity. Regarding A375 cell viability, a dose-dependent decrease was also observed after 24 h of treatment with PUE-ProN (Figure 9C), with a more pronounced reduction at the highest concentration. The viability values were: 88.40% at 25 µg/mL, 69.46% at 50 µg/mL, 64.91% at 75 µg/mL, 63.70% at 100 µg/mL, and 44.66% at 200 µg/mL.

Cytotoxic effects of PUE-formulations have also been described in the literature for different types of cancer. For example, Wang et al. evaluated the effect of a PUE nanosuspension on HT-29 colon cancer cells, alongside free PUE (at concentrations ranging from 1.25 to 25 µg/mL) over 24 h exposure time. Their results demonstrated that the nanosuspension exerted a stronger inhibitory effect on cell growth compared to free PUE [53].

In line with this observation, vesicular and other nanocarrier-based systems have frequently been reported to enhance the apparent bioactivity of poorly water-soluble phytochemicals by improving their effective cellular exposure compared with the free compound. Fahmy et al. reported that nanovesicles enhanced anti-melanoma performance and produced a stronger cytotoxic response in A375 cells compared with the corresponding free hydrophobic payload, while the drug-free (olive oil) niosomes showed no significant cytotoxicity, supporting a delivery-driven enhancement rather than excipient toxicity [54]. They attributed the increased cytotoxicity primarily to improved aqueous dispersibility of the hydrophobic payload upon encapsulation in the niosomal carrier [54,55]. The authors also highlighted that the use of non-ionic surfactants such as Span60 in formulation design has been associated with enhanced anticancer activity of the loaded compound in prior reports [54,56].

In addition, flavonoid-loaded niosome nanoparticles have been reported to exert notable anticancer effects, including substantial cytotoxicity and apoptosis-related responses in cancer cell models, supporting the concept that vesicular encapsulation can potentiate the biological action of poorly water-soluble phytochemicals [57]. More broadly, pro-/niosomal systems have been reviewed as versatile carriers in cancer-oriented drug delivery, with the potential to improve the effective exposure of encapsulated bioactive molecules by enhancing solubility, bioavailability and enabling carrier-mediated delivery [58]. Taken together, these literature findings support that the higher cytotoxic response observed for PUE-ProN versus free-PUE may plausibly reflect improved carrier-mediated delivery and cellular exposure at the same nominal concentration.

#### 2.4.2. Bright-Field Evaluation Morphological Assessment

*In vitro* cell morphology analysis provides essential information about cell behavior and functional status. Observation of shape, structure, and internal components allows the early identification of alterations and highlights how cells respond to different environmental factors. The results obtained show that morphological changes can reflect both cellular adaptation and potential pathological processes, confirming the value of this method as an effective and essential tool for interpreting cellular processes [59].

**Figure 10:**
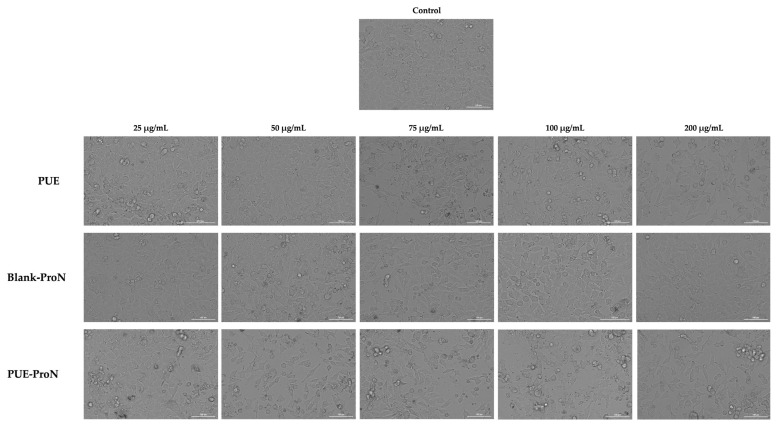
The morphological appearance of A375 cells after 42 h treatment with PUE, Blank-ProN, and PUE-ProN at 25, 50, 75, 100 and 200 µg/mL. The scale bar indicates 100 µm.

Morphological analysis (Figure 10) showed that treatment with the PUE compound induces changes in cell confluence, particularly at concentrations of 75 and 200 µg/mL. At these tested concentrations, cells showed a reduction in confluence compared to the control, which may suggest an inhibitory effect on cell viability. Mild concentration-dependent morphological changes were also observed, such as rounded or slightly retracted cells, as well as slight changes in cell shape.

In the case of Blank-ProN, no changes in cell morphology were observed. Treatment with F-Blank in the range of 25–200 µg/mL did not induce alterations in cell shape or appearance; even at the maximum concentration of 200 µg/mL, the cells appeared similar to control (untreated) cells, maintaining their normal morphology throughout the experiment. Similarly, exposure to PUE-ProN at concentrations ranging from 25 to 200 µg/mL resulted in a morphology largely equivalent to the control, the only notable change being a dose-dependent reduction in confluence.

#### 2.4.3. Mitochondrial and Nuclear Immunofluorescence Staining

Given the central role of the nucleus in critical cellular processes such as DNA replication, genome transcription, and protein synthesis, evaluating the nuclear response following exposure to different substances has particular importance. Morphological alterations of the nucleus (including changes in nucleus appearance, size, and chromatin organization) can serve as indicators of disturbances at this level and reflect the cellular stress induced by various treatments [60]. Hoechst 33342 is a fluorescent DNA-binding dye widely used to visualize nuclei in live and fixed cells. The dye binds within the minor groove of double-stranded DNA and emits a characteristic blue fluorescence. This fluorescence allows clear visualization of the nuclear contour and chromatin organization, enabling the detection of structural changes such as condensation of chromatin or nuclear fragmentation, which signal alterations in nuclear integrity [61].

In addition to nuclear assessment, visualization of internal structures, such as mitochondria, is important for both early identification of disorders and understanding of pathological processes. Literature reports indicate the involvement of mitochondria in metastasis and the development of resistance to therapeutic agents used in the treatment of various cancers, including melanoma [62]. The mitochondrial appearance is frequently analyzed using MitoTracker dyes. These fluorescent markers accumulate in active mitochondria in a membrane potential-dependent or independent manner, depending on the type of dye. Their ability to permanently bind to mitochondrial proteins allows for a clear and stable visualization of mitochondrial size and morphology. Due to their specificity, stability, and compatibility with other markers, MitoTrackers are a valuable tool for *in vitro* cellular studies [63]. A similar approach regarding the impact of PUE on cancer cells was reported by Zhang et al. in SMMC-7721 hepatocellular carcinoma cells, evaluating both nuclear and mitochondrial responses to treatment with PUE (0–1500 μg/mL at 24 h). The study showed pronounced nuclear morphological alterations, along with a significant decline in mitochondrial membrane potential [64]. Additionally, the technique used to visualize the appearance of the nuclei after exposing U251 and U87 (glioblastoma cells) to PUE for 48 h at a concentration of 200 micromolar also showed nuclear damage [65].

**Figure 11:**
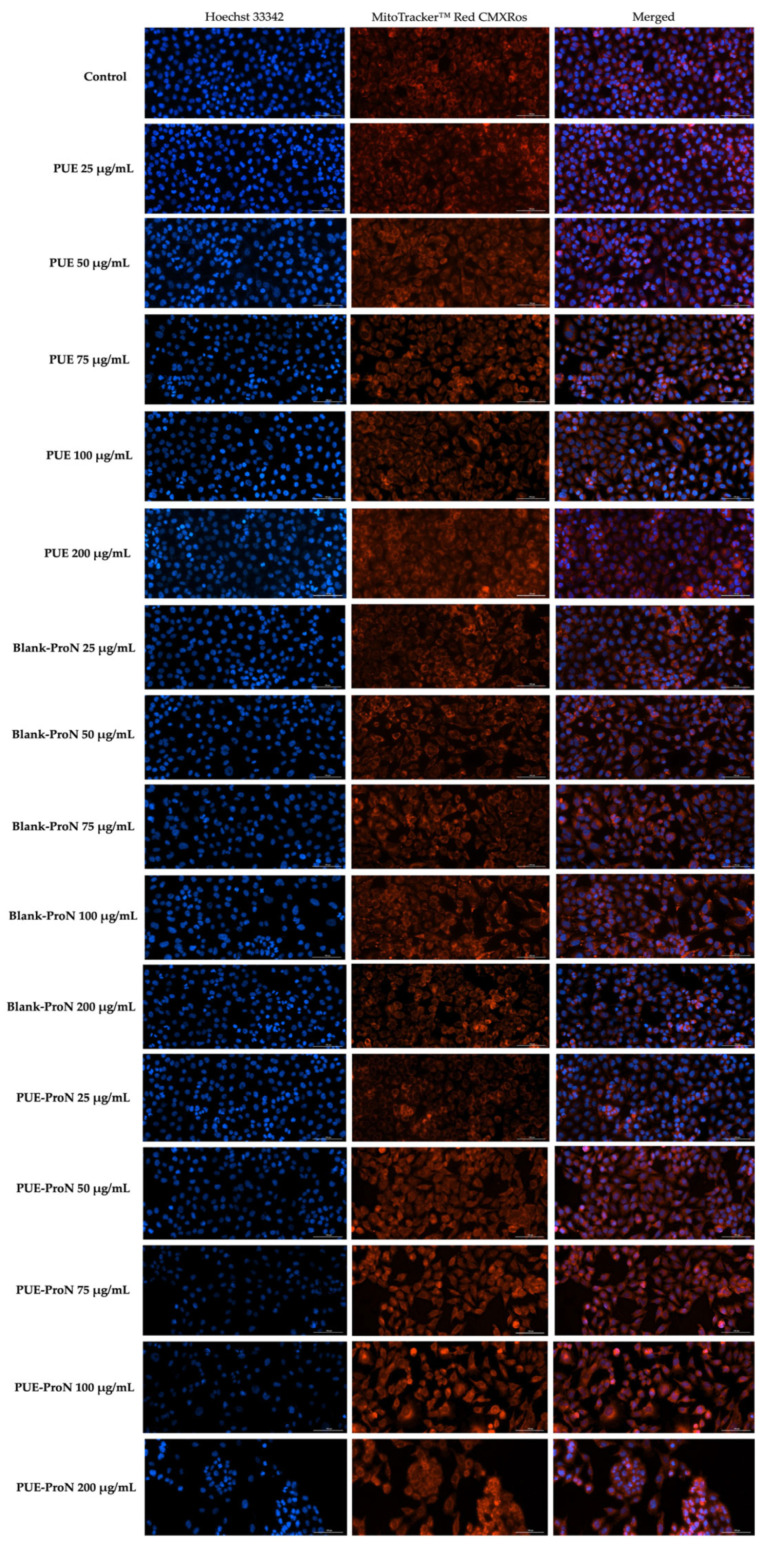
Representative images showing the mitochondrial and nuclear morphology of A375 cells following 24 h exposure to PUE, Blank-ProN and PUE-ProN at 25, 50, 75, 100, and 200 µg/mL. The scale bar indicates 100 µm.

Regarding the analysis of nuclear and mitochondrial morphology (Figure 11) after PUE treatment, the images obtained did not show any changes in the nuclei or mitochondrial structure, which retained their normal appearance, with no signs of chromatin condensation, changes in the shape of the nuclei, or visible alterations in mitochondrial architecture. These observations indicate that PUE did not induce structural changes in the nucleus or mitochondria at the concentrations tested.

For Blank-ProN, the nuclei retained a normal appearance at all concentrations tested, with no signs of chromatin condensation or changes in nuclear shape. At the mitochondrial level, although the overall morphology remained comparable to that of the control, a progressively enhanced punctate fluorescence was observed, without indicating mitochondrial structural disruption or fragmentation. Cell confluence remained similar to that of the control group under all conditions tested.

Regarding the nuclear and mitochondrial morphology following treatment with PUE-ProN, the images reveal structural alterations that correlate with the dose-dependent reduction in cell confluence. At concentrations of 25–50 µg/mL, both nuclei and mitochondria exhibited an appearance comparable to the control, without notable structural deviations. Beginning at 75 µg/mL, nuclear condensation became apparent, accompanied by localized increases in mitochondrial fluorescence. In parallel, the mitochondrial network gradually adopted a more rounded and fragmented appearance. These structural changes became progressively more pronounced at the highest concentration tested (200 µg/mL). Overall, the dose-dependent reduction in confluence, the nuclear and mitochondrial alterations observed at ≥75 µg/mL are consistent with the MTT viability decrease measured for PUE-ProN, supporting agreement between qualitative imaging endpoints and the metabolic viability readout.

### 2.5. In Ovo Irritant Evaluation via HET-CAM Assay

**Figure 12:**
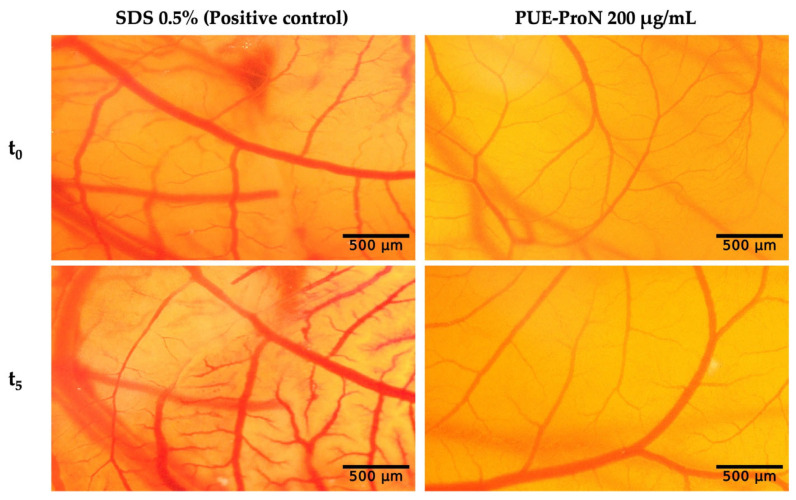
HET-CAM evaluation of SDS 0.5% and PUE-ProN (200 µg/mL). Stereomicroscope images show CAM vascular changes before (t_0_) to 300 s post-application (t_5_). Scale bar represents 500 µm.

Based on our previous *in vitro* studies, a concentration of 200 µg/mL was selected for further evaluation. The application of the PUE-ProN (200 µg/mL) did not induce any changes in the evaluated vascular parameters (Figure 12). In contrast to SLS 0.5% (positive control), which produced a high IS of 14.2 ± 0.14 (Table 9), PUE-ProN was well tolerated (IS = 0.43 ± 0.06) and exhibited no irritative effects on the CAM.

## 3. Conclusions

In this study, a Puerarin-loaded proniosomal gel (PUE-ProN) was successfully developed using, as ingredients, Span 60, Tween 80, phosphatidylcholine, and cholesterol. This composition provided physiologically acceptable pH (7.1 ± 0.12) and adequate entrapment efficiency (62 ± 0.26%). The formulation exhibited a polydisperse vesicular population (PDI 0.763 ± 0.17), in which the number-weighted profile showed a major vesicle population in the 100–200 nm range and a zeta potential of −34.9 ± 0.14 mV. Rheological profiling indicated non-Newtonian, pseudoplastic and thixotropic behavior, favorable for topical application. From a biological perspective, the gel vehicle was non-cytotoxic, while PUE-ProN induced a concentration-dependent reduction in A375 viability, reaching 44.66% at 200 µg/mL, and produced dose-associated nuclear condensation and mitochondrial network disruption at higher concentrations. Moreover, PUE-ProN was classified as non-irritant *in ovo* (IS = 0.43 ± 0.06). Together, these findings support PUE-ProN as a promising topical candidate platform with *in vitro* antimelanoma potential and favorable preliminary tolerability, but further investigation is required.

The key limitations of the present study are the absence of *in vitro* release and *ex vivo* permeation/retention data, as well as the lack of internal gel microstructure imaging, which are required to fully substantiate dermal delivery performance. Future work should include (i) *in vitro* release profiling, together with *ex vivo* skin permeation/retention and spreadability assessment, to confirm dermal delivery performance; (ii) long-term stability assessment; and (iii) *in vivo* efficacy and safety studies to validate therapeutic relevance and translational potential.

## 4. Materials and Methods

### 4.1. Reagents

Puerarin (CAS No. 3681-99-0; product code MP08125) was obtained from BYOSINTH (Bratislava, Slovakia, 2023). Ethanol, sodium dodecyl sulfate (SDS) and the lipid components (e.g., sorbitan monostearate (Span 60), polyoxyethylene sorbitan monooleate (Tween 80), phosphatidylcholine, cholesterol) were purchased from Sigma-Aldrich, Merck KGaA (Darmstadt, Germany). The MTT assay kit (3-(4,5-dimethylthiazol-2-yl)-2,5-diphenyltetrazolium bromide) and phosphate-buffered saline (PBS), both obtained from Sigma-Aldrich, Merck KGaA (Darmstadt, Germany). Dulbecco’s Modified Eagle Medium (DMEM) and the penicillin/streptomycin solution were purchased from PAN-Biotech GmbH (Aidenbach, Germany).

### 4.2. Formulation and Characterization of the Proniosomal Gels (Blank-ProN, PUE-ProN)

The proniosomal gels were prepared using the coacervation-phase separation method [36,66,67], employing a combination of nonionic surfactants and phospholipids. The lipid components (e.g., Span60, Tween80, phosphatidylcholine, cholesterol) were accurately weighed in predetermined ratios and dissolved in absolute ethanol in a glass vial at 50 °C under continuous stirring until a clear, and homogeneous solution was obtained. To minimize ethanol evaporation, the vial was covered during heating and stirring. Preheated Milli-Q water was used to dissolve the isoflavone PUE separately. Subsequently, the PUE solution was incorporated to the mixture in small amounts and stirred gently for 5 min. A yellow viscous, creamy, translucent gel gradually formed upon cooling to room temperature, indicating the successful formation of the gel. The prepared gel was stored in airtight containers, protected from light and moisture, until further characterization.

#### 4.2.1. pH and Entrapment Efficacy Measurements

The pH was determined using a digital pH meter, following the established protocol: 1 g of gel was dispersed in 10 mL of Milli-Q water, and the measurement was performed in triplicate.

For the determination of entrapment efficiency, 1 g of the gel sample was dispersed in 10 mL of Milli-Q water. The dispersion was then clarified by filtration to remove coarse particulate matter, followed by centrifugation at 10,000 rpm for 15 min at 4 °C. The supernatant was analyzed using UV-Visible spectrophotometry (UviLine 9400 Spectrophotometer, SI Analytics, Deutschland, Germany), and the absorbance was measured at 260 nm.

(1) $$Entrapment\;Efficiency\;\left(EE\%\right)=\left(\;\frac{W_{initial}-W_{free}}{W_{initial}}\;\right)\times 100$$

where $W_{initial}$ indicates the theoretical amount of the PUE in the formulation and $W_{free}$ the amount of the PUE in the supernatant. These values were quantified by UV–Visible spectrophotometry at 260 nm using a calibration curve for PUE (y = 581.34x + 0.1976; R^2^ = 0.9993).

#### 4.2.2. Fourier Transform Infrared (FTIR) Analysis

FTIR spectra of raw materials were recorded using potassium bromide (KBr) pellet techniques under reduced pressure. For PUE-ProN, the spectrum was acquired in transmission mode using IR-transparent windows (KBr plates): a small amount of gel was spread as a thin, uniform film between two IR windows and mounted in the sample holder to obtain the spectrum. All spectral measurements were conducted at ambient temperature (25 °C), acquired in the spectral range of 4000–400 cm^−1^, utilizing a Jasco FTIR spectrophotometer (Jasco, Tokyo, Japan).

#### 4.2.3. Particle Size and Zeta Potential Analysis

Dynamic light scattering (DLS) measurements were performed using a Zetasizer Nano ZS (Malvern Panalytical Ltd., Malvern, UK) to determine the hydrodynamic diameter and zeta potential of the gel formulations. Proniosomal gels were hydrated by dispersing 1 g gel in 10 mL Milli-Q water (final concentration: 100 mg/mL) under gentle vortexing until a homogeneous dispersion was obtained, followed by equilibration for 5 min at room temperature. The analysis was conducted at a controlled temperature of 25 ± 2 °C using non-invasive backscatter (NIBS) detection at 173°. All measurements were performed in triplicate (*n* = 3), and the results are reported as mean ± SD.

#### 4.2.4. Differential Scanning Calorimetry (DSC) Analysis

Blank-ProN and PUE-ProN were analyzed using Netzsch DSC 204 F1 Phoenix (Selb, Germany) equipment. An empty sealed (crimped) aluminum crucible was used as a reference. Samples were placed in sealed (crimped) aluminum crucibles and heated with 10 Kmin^−1^ from 25 °C to 150 °C in an inert nitrogen atmosphere.

#### 4.2.5. Rheological Analysis

Rheological properties of PUE-ProN were evaluated using a Brookfield AMETEK DV2T rheometer (AMETEK Brookfield, Middleborough, MA, USA). Measurements were performed in a temperature-controlled setup with a fixed outer cylinder and a rotating inner cylinder (SC4-21) operating at predefined speeds. The device measures the torque generated by the annular layer of material introduced between the two cylinders.

Experimental determinations were performed at 25 ± 0.5 °C and 37 ± 0.5 °C, at the shear rate ramp-up (from 0.93 s^−1^ to 93 s^−1^ for 180 s) and ramp-down (from 93 s^−1^ to 0.93 s^−1^ for 180 s). Herschel–Bulkley model was used to describe the rheological properties, defined by Equation (2) [68,69]:

(2) $$\tau =\tau_{0}+k\cdot {\dot{\gamma }}^{n}$$

where τ and $\tau_{0}$ are the shear stress and the yield stress, respectively, *k*—consistency index, and *n*—flow index. The experimental data were fitted to the Herschel–Bulkley equation using the software package Table Curve 2D (Version 5.01).

### 4.3. Computational Studies

The molecular geometry of PUE was optimized at the B3LYP/6-311G level of theory using the Gaussian 09W software (Version 9.0) [70] package, both in the gas phase and in aqueous solution employing the IEFPCM solvation model. Vibrational frequency analysis confirmed that the optimized geometries correspond to true minima on the potential energy surface, and these structures were subsequently used for further calculations. The geo-metric characterization of the gas-phase and aqueous optimized isoflavone was obtained by means of Chem3D software (V20.1). The steric parameters, Connolly Accessible Area (CAA), Connolly Solvent-Excluded Volume (CSEV), and ovality, have been computed with Chem3D software. The melanoma antitumor activity of PUE was evaluated by molecular docking calculations. A corresponding grid box of 40 × 40 × 40 Å was used for each receptor, the center of the grid box being considered the center of the protein. The gas and aqueous optimized geometry of PUE was loaded as ligands, and all the torsions along the rotatable bonds were assigned. AutoDock Vina (V1.2.7) [71] has been used to obtain the binding affinities of PUE towards the two receptors, as well as for the visualization of their interactions.

### 4.4. In Vitro Evaluation

#### 4.4.1. Cell Culture Conditions

The A375 human malignant melanoma cell line (CRL-1619™; ATCC, Manassas, VA, USA) was used in this study. The cells were cultured in Dulbecco’s Modified Eagle Medium (DMEM) supplemented with 10% fetal bovine serum (FBS), 1% penicillin/streptomycin solution, and maintained at 37 °C in a humidified atmosphere containing 5% CO_2_.

#### 4.4.2. Reagents and Instruments

Hoechst 33342 dye and MitoTracker™ Red CMXRos were supplied by ThermoFisher Scientific (Waltham, MA, USA). Cell viability and imaging analyses were performed using the Cytation 5 microplate reader and the Lionheart FX automated microscope, both from BioTek Instruments Inc. (Winooski, VT, USA). Morphological changes in A375 cells were evaluated using brightfield microscopy at 20× magnification on a Lionheart FX automated system. Representative images were obtained using the Lionheart FX automated microscope (20×) and processed using Gen5™ Microplate Data Collection and Analysis software (v3.14, BioTek Instruments Inc., Winooski, VT, USA).

#### 4.4.3. In Vitro Experimental Setup

The A375 human melanoma cells were treated with various test samples, including PUE, Blank-ProN and PUE-ProN, at concentrations of 25, 50, 75, 100, and 200 µg/mL. Stock solutions were prepared at 50 mg/mL and diluted in culture medium to achieve the experimental concentrations. Following 24 h of exposure, the cytotoxic profile and morphological changes were assessed using complementary assays. The MTT assay was employed to evaluate cell viability and determine the cytotoxic profile of the tested compounds. Hoechst 33342 and MitoTracker™ Red CMXRos staining were used to assess nuclear integrity and mitochondrial function, respectively. Cell morphology was examined microscopically to identify potential alterations in cell shape and density. All experiments were performed in triplicate to ensure reproducibility and statistical reliability.

#### 4.4.4. Cell Viability Assessment Through MTT Assay

The cytotoxic effect of PUE, Blank-ProN and PUE-ProN on A375 cells was evaluated using the MTT viability assay. A375 cells were seeded in 96-well plates at a density of 1 ×10^4^ cells/well and allowed to adhere until reaching approximately 80% confluence. After treatment with the test compounds at concentrations of 25, 50, 75, 100, and 200 µg/mL, the culture media were replaced, and 10 µL of MTT reagent was added to each well. Plates were incubated for 3 h at 37 °C in a 5% CO_2_ atmosphere. Then, 100 µL of MTT solubilizing solution was added per well, and the plates were incubated for 30 min at room temperature in the dark. Absorbance was measured at 570 and 630 nm using a Cytation 5 plate reader.

#### 4.4.5. Bright-Field Evaluation of Cell Morphology

To evaluate the effects of samples on the cell morphology of A375 cells, the cells were seeded in 96-well plates at a density of 1 × 10^4^ cells/well. Cells were allowed to grow until they achieved 80% confluence, after which they were treated with the test compounds at concentrations of 25, 50, 75, 100, and 200 µg/mL for 24 h.

#### 4.4.6. Evaluation of Mitochondrial and Nuclear Alterations

To evaluate mitochondrial and nuclear morphology, A375 cells were seeded in 12-well culture plates (1 × 10^5^ cells/well) and allowed to reach optimal confluence. They were then exposed for 24 h to different test samples (e.g., PUE, Blank-ProN, PUE-ProN) at the above-mentioned concentrations (25, 50, 75, 100, and 200 µg/mL). To visualize the mitochondrial network, the cells were stained with MitoTracker (300 nM), obtained by diluting the stock solution (1 mM in DMSO) in the complete culture medium. After 30 min of incubation under standard conditions, the cells were washed to remove excess dye and then fixed with 4% paraformaldehyde for 10 min at room temperature. For analysis of nuclear modifications, Hoechst 33342 solution (1:2000 in PBS) was added after fixation, with an incubation period of 5–10 min in the dark. The cells were then washed three times with PBS to remove unbound dye.

### 4.5. In Ovo Safety Evaluation

The preliminary biocompatibility profile of PUE-ProN was evaluated through an *in ovo* experiment using the HET-CAM assay, which evaluated its potential to cause local irritation. In our laboratory, we performed *in ovo* experiments using embryonated chicken eggs according to the methodology used in previous studies [72,73]. Local irritation potential was evaluated using 0.5% sodium dodecyl sulfate (SDS) (Sigma-Aldrich, Merck KGaA, Darmstadt, Germany) as a positive reference. A 5 Min stereomicroscope assessment (Discovery 8 stereomicroscope equipped with an Axio CAM 105 color camera; Zeiss, Göttingen, Germany) was conducted to detect possible alterations of the vascular plexus (Sec_H_, Sec_LS_, Sec_CG_). All images were processed using ImageJ version 1.48q software (U.S. National Institutes of Health, Bethesda, MD, USA, https://imagej.nih.gov/ij/index.html, accessed on 29 October 2025). Using Equation (3), we calculated an irritation score (IS) and classified it based on Luepke’s criteria (e.g., non-irritant 0÷0.9; weak irritant 1.0÷4.9; moderate irritant 5.0 ÷ 8.9; strong irritant 9.0 ÷ 21.0) [74].

(3) $$Irritation\;score\;\left(IS\right)=\left(5\times \frac{301-{Sec}_{H}}{300}\right)+\left(7\times \frac{301-{Sec}_{LS}}{300}\right)+\left(9\times \frac{301-{Sec}_{CG}}{300}\right)$$

### 4.6. Statistical Analysis

Statistical analyses were performed using GraphPad Prism version 10.5.0 (GraphPad Software, San Diego, CA, USA). One-way ANOVA and Dunnett’s multiple comparison test were used to assess differences between the different treatment groups (PUE, Blank-ProN, PUE-ProN) and the control. The statistically significant outcomes were determined using “*”: * *p* ≤ 0.05, ** *p* < 0.01, *** *p* ≤ 0.001, and **** *p* ≤ 0.0001.

## Figures and Tables

**Table 1:** The composition of the PUE-loaded proniosomal gel formulation.

Components	Composition
PUE	3% *w*/*w*
Span60	30 mg
Tween80	150 mg
Cholesterol	30 mg
Phosphatidylcholine	90 mg
Absolute ethanol	0.3 mL
Milli-Q water	0.1 mL

**Table 2:** The values of rheological parameters for PUE-ProN.

Parameter	Temperature, °C	
	25 ± 0.5	37 ± 0.5
**Steady state viscosity, Pa.s**	0.333 ± 0.008	0.136 ± 0.003
**Thixotropy, Pa/s**	145.85 ± 0.004	41.93 ± 0.011

**Table 3:** Herschel–Bulkley parameters for PUE-ProN.

Temperature/Shear Rate	$\tau_{0}$ (Pa)	k ($Pa\cdot s^{n}$)	*n*	R^2^
**25 °C/up**	0.854	0.720	0.819	0.99985
**25 °C/down**	1.200	0.330	0.979	0.99994
**37 °C/up**	0.289	0.270	0.839	0.99986
**37 °C/down**	0.208	0.197	0.906	0.99970

**Table 4:** Steric parameters of PUE.

Compound	CAA (Å^2^)	CSEV (Å^3^)	Ovality
**PUE (gas)**	640.293	327.366	1.511
**PUE (aq.)**	643.473	327.563	1.517

**Table 5:** Binding affinities (for the best nine conformations) between PUE, ML329 and MITF.

Compound	Binding Affinities (kcal/mol)								
	E1	E2	E3	E4	E5	E6	E7	E8	E9
**PUE (gas)**	−8.3	−8.3	−8.2	−8.2	−8.1	−7.8	−7.7	−7.7	−7.7
**PUE (aq.)**	−8.4	−8.3	−8.2	−8.1	−8.0	−7.9	−7.7	−7.7	−7.7
**ML329**	−8.1	−7.6	−7.4	−6.9	−6.6	−6.4	−6.3	−6.3	−6.1

**Table 6:** Interactions PUE (ligand)—receptor MITF.

Compound	Hydrogen Bonds	Atoms in Close Contact Interactions
**PUE (gas)**	2.889 Å (3″-OH-DT16)	Arg240; DC15 (chromene) DG2 (sugar residue) DA14; DG5 (phenyl)
**PUE (aq.)**	2.917 Å (4″-OH-DT16)	Arg240; DC15 (chromene) DG2 (sugar residue)
**ML329**	3.045 Å (DT16)	Arg240; DC15; DA14; DA4; DT3

**Table 7:** Binding affinities (for the best nine conformations) between PUE, 5-azacytidine and DNMT3B.

Compound	Binding Affinities (kcal/mol)								
	E1	E2	E3	E4	E5	E6	E7	E8	E9
**PUE (gas)**	−8.4	−8.1	−7.8	−7.4	−7.3	−6.9	−6.9	−6.9	−6.8
**PUE (aq.)**	−8.5	−8.0	−8.0	−7.4	−7.4	−7.4	−7.0	−7.0	−6.9
**5-azacytidine**	−5.9	−5.8	−5.8	−5.7	−5.7	−5.7	−5.6	−5.5	−5.5

**Table 8:** Interactions PUE (ligand)—receptor DNMT3B.

Compound	Hydrogen Bonds	Atoms in Close Contact Interactions
**PUE (gas)**	-	**Ile233, Pro38** (chromene cycle) **Val35**, Trp260, His304 (phenyl ring) **Lys276**, Ser273, Met258, Glu307, Val311 (sugar residue)
**PUE (aq.)**	-	**Ile233, Pro38**, His304 (chromene cycle) **Val35**; Lys251; Trp260 (phenyl ring) **Lys276**; Ser273; Glu307; Val311 (sugar residue)
5-azacytidine	3.135 Å (Glu271)	**Glu307; Lys308**

**Table 9:** Irritation scores and type effects of positive control and PUE-ProN.

Samples	Irritation Score (IS)	Type of Effect
**SDS 0.5%** **(positive control)**	14.2 ± 0.14	Strong irritant
**PUE-ProN 200 µg/mL**	0.43 ± 0.06	Non-irritant

## Data Availability

The original contributions presented in the study are included in the article; further inquiries can be directed to the corresponding author.
